# Impact of Mechanical Arc Oscillation on the Microstructure and Durability of Welded Joints in Molten Salt Thermal Storage System

**DOI:** 10.3390/ma18071619

**Published:** 2025-04-02

**Authors:** Raúl Pastén, Mauro Henríquez, Mehran Nabahat, Victor Vergara, Juan C. Reinoso-Burrows, Carlos Soto, Carlos Durán, Edward Fuentealba, Luis Guerreiro

**Affiliations:** 1 Centro de Desarrollo Energético Antofagasta, Universidad de Antofagasta, Angamos 601, Antofagasta 1240000, Chile; raul.pasten@uantof.cl (R.P.); juan.reinoso.burrows@ua.cl (J.C.R.-B.); carlos.soto@uantof.cl (C.S.); carlos.duran@uantof.cl (C.D.); edward.fuentealba@uantof.cl (E.F.); 2 Iberian Centre for Research in Energy Storage (CIIAE), 10003 Cáceres, Spain; mehran.nabahat@ciiae.org; 3 Departamento de Ingeniería Mecánica, Universidad de Antofagasta, Av. Universidad de Antofagasta, Antofagasta 1240000, Chile; victor.vergara@uantof.cl (V.V.); lguerreiro@uevora.pt (L.G.); 4 ICT—Institute of Earth Sciences, University of Évora, 7000-308 Évora, Portugal

**Keywords:** solar salt, stainless steel, welded junctions, corrosion, oscillating arc welding, dilution

## Abstract

The two-tank molten salt thermal storage system is the most common storage solution in concentrated solar power (CSP) plants. Solar salt (60% NaNO_3_ + 40% KNO_3_) is the most widely used energy storage material in solar thermal plants. In solar tower technology, where the molten salts must operate at temperatures ranging from 290 °C to 565 °C, several issues related to tank failures have emerged in recent years, with some of these failures attributed to the welding process. The welding process of joints in 316L stainless steel (ASS) probes exposed to a moving flow of a binary mixture containing 60% NaNO_3_ and 40% KNO_3_ (solar salt) is analysed. The results were evaluated using scanning electron microscopy (SEM) at 120, 500, 1000, 1500, and 2300 h of exposure. It was identified that arc mechanical oscillations significantly improve the microstructural properties and geometrical characteristics of welded joints, reducing structural defects and improving corrosion resistance. The technique promotes uniform thermal distribution, refined dendrite morphology, and homogeneous alloying element distribution, resulting in lower mass loss in high-temperature molten salt environments. Additionally, oscillation welding optimises the bead geometry, with reduced wetting angles and controlled penetration, making it ideal for high-precision industrial applications and extreme environments, such as molten salt thermal storage systems.

## 1. Introduction

Solar tower concentrated solar power (CSP) plants use two-tank thermal storage systems for molten solar salts: one “cold” salt tank and one “hot” salt tank. In the case of parabolic trough technology, the cold salt tank typically operates at temperatures ranging from 290 °C to 393 °C [1]. In tower technology, the hot salt tank can reach temperatures of up to 565 °C. The capacity of these tanks is directly related to the plant’s installed power, allowing for the storage of thousands of tons of solar salts. This system ensures energy supply during periods without solar radiation, maximising the continuous operation of the plant. The thermal energy storage (TES) system is a fundamental component of concentrated solar power (CSP) plant technology [2]. Currently, the most commonly used material in solar thermal plants is solar salt (60% NaNO_3_ + 40% KNO_3_) [3,4,5,6].

Regarding the materials used in tank construction, they are designed to withstand the thermal and chemical conditions induced by molten salts. Stabilised stainless steels, such as AISI 347H and AISI 321H, are used for hot salt tanks, offering high corrosion resistance at elevated temperatures [7,8]. On the other hand, the cold salt tanks are typically made of carbon steel ASTM A516 Gr-70, a material known for its high pressure resistance, or stainless steels such as AISI 304. These material selections are optimised to ensure the tanks’ structural integrity and service life under the operational conditions of CSP plants [9,10]. The tanks are insulated with mineral wool, refractory bricks, or ceramic materials to reduce heat losses. Due to their large dimensions, these systems are manufactured onsite at the plant [11]. The tanks are equipped with piping systems for the inlet and outlet of molten salts, pumps, and high-pressure valves capable of withstanding extreme temperatures. The tanks typically have a flat bottom and a dome-shaped cover. The floor and shell thicknesses are designed with minimum values based on the API 650 standard. Although there is not yet a specific technical standard for the design of TES tanks with molten salts, current tanks are designed according to API 650 and ASME Section II standards, which are limited for the high temperatures and conditions of TES tanks with molten salts [12].

Although the molten salts have been successfully used in parabolic trough plants, failures have been reported in the tanks of central receiver plants, causing economic losses and scepticism about the technology [9,13]. These failures are related to the early stage of the technology and issues in design, manufacturing, commissioning, and operation. A representative example is the CSP Crescent Dunes project in the United States of America. The problems associated with the molten salt tank in this project included issues such as corrosion, operational reliability, thermal stress, maintenance challenges, and cost implications. These problems highlight the importance of meticulous design and the proper selection of materials in molten salt tank systems for concentrated solar power applications [13].

Among the failures of storage tanks, there remains significant uncertainty regarding the evaluation of welded joints in contact with the molten salts at high temperatures [14]. Welding thermal storage tanks, particularly those used in CSP plants with molten salt energy storage systems, is a critical process to ensure the structural integrity and operational safety of these systems. These tanks are subjected to high temperatures, with molten salts reaching up to 565 °C, creating a highly extreme environment where both materials and welding techniques must be carefully selected. Currently, the most commonly used welding processes in storage systems are gas metal arc welding (GMAW) and gas tungsten arc welding (GTAW), both of which require careful control to minimise defects and ensure long-term durability [9,14,15].

In general, specific studies on welded joints in thermal storage tanks are limited, particularly with respect to the behaviour of fusion zones (FZs) and heat-affected zones (HAZs). In these regions, intergranular corrosion, segregation of interdendritic carbides, loss of passivation in grain cores, and grain size modifications are expected to occur, which may favour stress corrosion cracking (SCC) and stress relaxation cracking (SRC). In addition, the chemical interaction of elements with molten salts and the transformation of delta ferrite to the sigma (σ) phase represent additional challenges for the structural integrity of welded joints.

Post-treatment strategies to mitigate these effects have been evaluated in several studies. For example, the behaviour of a weld bead on an AISI 316L steel substrate was compared with and without laser post-treatment [7]. The results indicated that the application of a laser hypertempering significantly reduced intergranular corrosion, suggesting that this surface treatment contributes to relieving residual stresses and dissolving precipitated carbides. Similarly, Austin and Ruiz [16] recommended the application of a post-weld heat treatment (PWHT) to relieve residual stresses, improve fatigue resistance, and control hardness in the HAZ, thereby reducing the risk of failure.

Surface coatings have been considered an effective alternative to mitigate the effects of direct contact with corrosive media in welded zones. In this context, various thermal spraying techniques, such as Arc Spraying (AS) and High-Velocity Arc Spraying (HVAS), have been investigated. For example, Pombo Rodríguez et al. [17], Buchanan et al. [18], and Amushahi et al. [19] have conducted studies on different coating applications and their performance under various conditions. In addition, Xu et al. [20] analysed the application of a PWHT on an electric-arc-applied Mg-Al coating on welded parts, concluding that such a coating provides effective corrosion protection. Likewise, Kondaiah et al. [21] explored fractal coatings, developing an innovative model based on nickel electrodeposition on austenitic stainless steels (ASSs). Their study demonstrated the formation of Ni oxides on the surface, acting as passive layers that inhibited corrosion.

Regarding dissimilar filler materials (FMs), Ward et al. [22] focused on the welding behaviour of type 347 ASS exposed to high temperatures in nuclear applications. the study concluded that the filler metal ER16-8-2 has significant advantages compared to other weld alloys in stainless steel due to its ability to generate joints with minimal delta ferrite in the microstructure.

Austenitic stainless steels (ASSs) contain at least 10.5% chromium, which allows for the formation of a thin passivating layer of Cr_2_O_3_ with a thickness of 1 to 3 nm, which effectively protects against corrosion in various oxidising and reducing environments [23]. In addition, nickel, present in concentrations of 8% to 10%, plays a crucial role in inhibiting the precipitation of chromium carbides (Cr_23_C_6_) at grain boundaries, preventing the depassivation of grain nuclei [24].

Finally, no previous studies have been found that examine the use of oscillated arc welding in stainless steel storage tanks for thermal energy storage systems. Therefore, this study represents the first one to be conducted in a pilot plant with molten salts operating at 400 °C. To address this gap, the Center for Energy Development at the University of Antofagasta (CDEA), in collaboration with the Iberian Center for Research in Energy Storage (CIIAE), is carrying out a study to evaluate the influence of mechanical arc oscillation on weld bead formation using the GMAW process with short-circuit metal transfer in a high-temperature molten salt environment.

## 2. Materials and Methods

### 2.1. Materials

In this study the influence of mechanical arc oscillation was evaluated on the morphology and dilution of weld beads using the GMAW welding process, comparing the results with welds performed without oscillation. Immersion tests were conducted on stainless steel (316L) samples exposed to liquid solar salt at a constant temperature of 400 °C. The experiment aimed to assess how prolonged exposure to high temperatures affects the properties of welded joints and their corrosion resistance. The solar salt nitrates were prepared using sodium nitrate (NaNO_3_, 99.5%) and potassium nitrate (KNO_3_, 99.5%), both supplied by SQM (Antofagasta, Chile).

### 2.2. Welding Process

The plate on which the welding processes were carried out consists of an austenitic stainless steel (ASS) of type 316L with dimensions of 250 × 150 × 6 mm. Its chemical composition is shown in Table 1.

**Figure 1:**
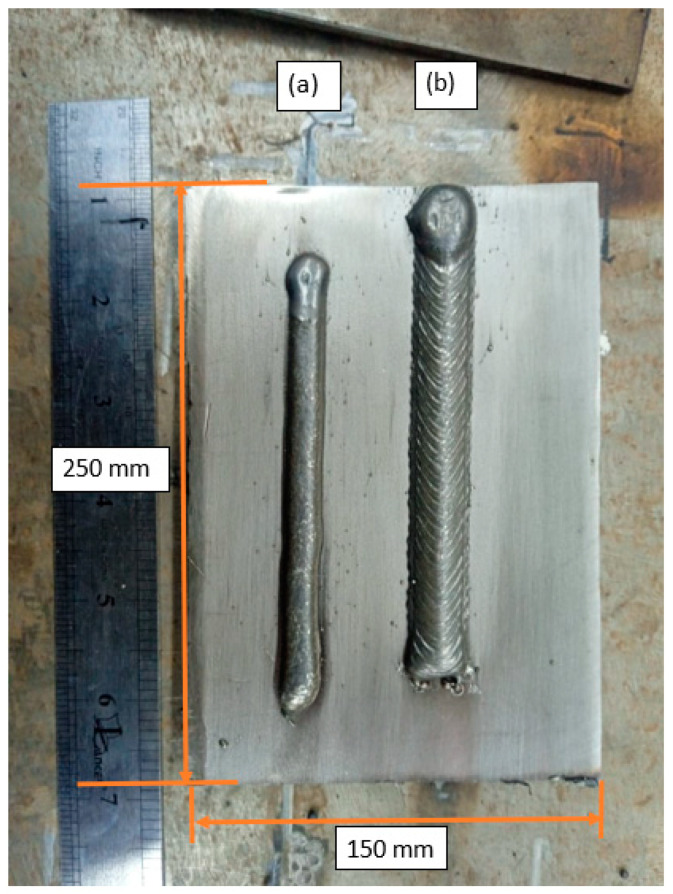
Weld beads (**a**) without oscillations; (**b**) with oscillations.

To evaluate the performance of both welding processes, two weld beads were created on an AISI 316L steel plate (Küpfer Hermanos S.A., Antofagasta, Chile) measuring 250 × 150 × 6 mm (Figure 1) using a similar filler material (FM), ER316L, through a GMAW process with short-circuit metal transfer, with and without mechanical oscillation of the electric arc (Figure 1).

**Figure 2:**
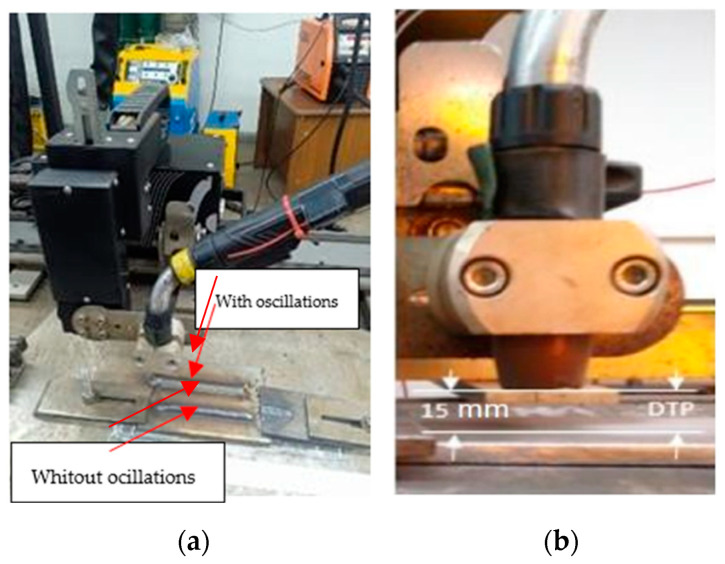
(**a**) Tartilope V4 CNC welder that performs mechanical oscillations adapted to a gun for the GMAW process. (**b**) Distance from the sample nozzle.

The welding process was carried out using a CNC Tartilope V4 welder (SPS, Florianópolis, Brazil) (Figure 2a), with four degrees of freedom for the torch, generating a triangular oscillatory motion of the electric arc for one of the weld beads. The second bead was created without mechanical arc oscillation. This CNC Tartilope V4 equipment provides full control and repeatability of parameters such as frequency, lateral stop time, and oscillation amplitude.

A shielding gas mixture of 80% argon and 20% carbon dioxide (80% Ar + 20% CO_2_) was used, with a fixed flow rate of 16 L/min, measured using a gas mixer. The torch was aligned vertically at 90° to the base material (BM), allowing for easy adjustment of the contact-tip-to-workpiece distance (DTP) (Figure 2b).

The welding parameters used are shown in Table 2. This matrix comprises the wire speed, oscillation frequency, and type of oscillations. From this, constant wire speeds of 4 m/min and a frequency of 1 Hz for the oscillating arc were selected, while, on the other hand, an electric arc without oscillation was used.

The parameters that were kept constant for manufacturing both welding beads are shown in Table 2. The frequency used for the bead with oscillations was 1 Hz and 0 Hz for welding without oscillations (Table 3).

### 2.3. Procedure for Determining Welding Current and Arc Energy

**Figure 3:**
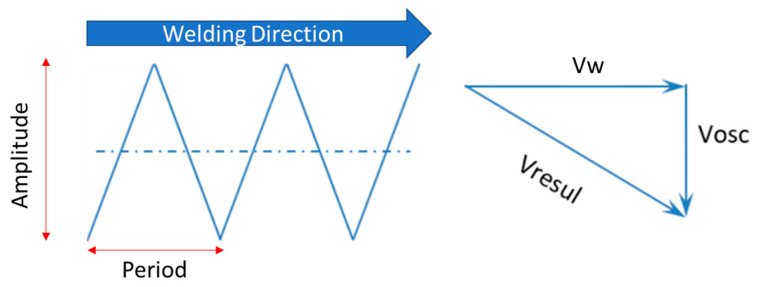
Resulting welding speed using the weaving technique. $V_{w}$: welding speed, $V_{osc}$: oscillations speed and $V_{resul}$: resulting speed.

**Figure 4:**
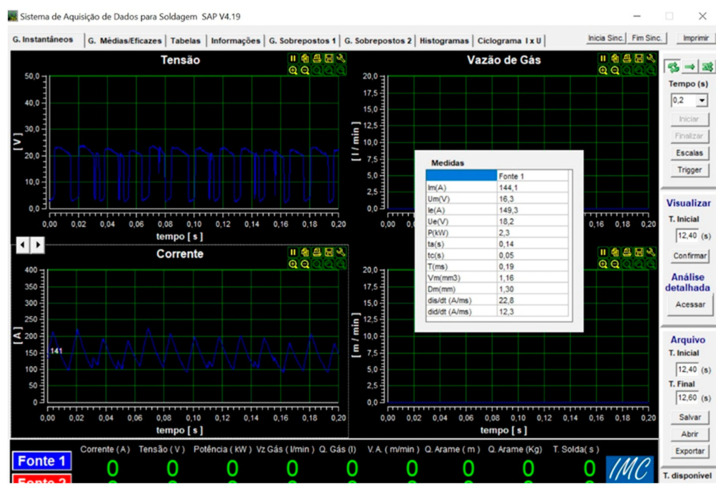
The data acquisition system SAPV4.

The welding energy ($E_{w}$) is determined using the weaving technique, which consists of a lateral movement of the electrode or torch to improve fusion and weld bead filling, optimising heat transfer and deposit quality [25,26]. To calculate it, the following equation is used:

(1) $$E_{w}=\frac{\frac{\sum \mathrm{Ui}*\mathrm{Ii}}{n}}{V_{\mathrm{resul}}}=\frac{\frac{\sum \mathrm{Pi}}{n}}{V_{\mathrm{result}}}*600\;J/\mathrm{mm}$$

where V_resul_ (mm/min) is the resulting speed (Figure 3); I_i_ is the instantaneous welding current (A); U_i_ is the instantaneous arc voltage (V); and Pi is the instantaneous arc power (kW) and the result of the product “Ui ∗ Ii”. The welding current and arc power Pi are obtained from the data acquisition system, as shown in Figure 4, available in the welding laboratory.

The resulting welding speed for the oscillated weld was determined using the following equation [27]:

(2) $$V_{\mathrm{resul}}=\sqrt{\left({V_{w}}^{2}+{V_{\mathrm{osc}}}^{2}\right)}$$

where $V_{w}$ is the welding speed (cm/min) and $V_{osc}$ is the oscillation speed (cm/min), calculated based on oscillation amplitude and frequency. It should be considered that the transverse oscillation of the arc introduces an additional lateral speed to the weld pool, modifying its total speed. As a result, an equation relates the oscillation amplitude (A_osc_) and the oscillation frequency (f) to the oscillatory speed (mm/min) ($V_{osc}$), as can be seen in Equation (3).

(3) $$V_{\mathrm{osc}}=A_{\mathrm{osc}}*f*\;\left(\frac{1}{10}\right)$$

where V_osc_ is the oscillation speed (cm/s); $A_{osc}$ is the oscillation amplitude (mm); f is the oscillation frequency (Hz); and $\left(\frac{1}{10}\right)$ is the unit conversion factor.

Table 4 shows the resulting welding speed (V_resul_), welding current (I_w_), and welding energy ($E_{w}$) obtained from the tests performed under two conditions: (a) without oscillation and (b) with oscillation.

There is a notable difference between the arc energies of welds with and without oscillations. In the first case, it reaches 0.188 (kJ/mm) compared to 0.960 (KJ/mm). This difference highlights the significant impact of the oscillating arc technique, which can influence the microstructure and mechanical properties.

### 2.4. Welding Filler Material

The filler material used was AWS ER-316L wire with a diameter of 1.2 mm, operated at a current range of 155–450 A and a voltage range of 20–34 V. This filler material provides a balanced chemical composition, resulting in uniform properties of the deposited metal and well-balanced mechanical properties. Its chemical composition and characteristics are shown in Table 5.

### 2.5. Preparation of Welded Specimens

**Figure 5:**
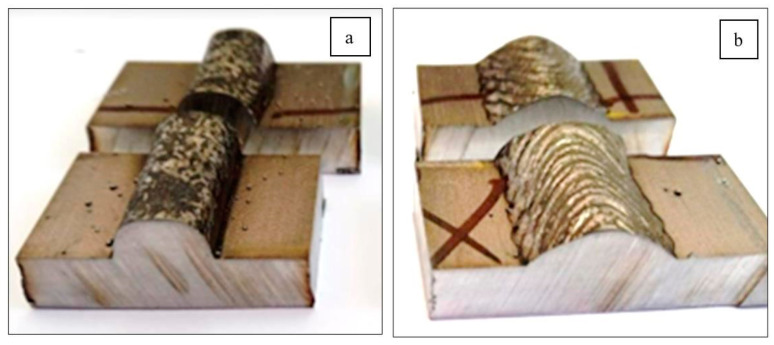
Stainless steel specimens with welding (**a**) without oscillations and (**b**) with oscillations.

Once the two types of weld beads were created, the plate was cut longitudinally and transversely using a Mecatome T260 cold cutting machine (MCS, Miami, FL, USA) to prepare four sets of specimens, each approximately 50 × 20 × 6 mm (5a without oscillations and 5b with oscillations), as shown in Figure 5. Each specimen was weighed using a BOECO balance, Model BAS 31 plus (Boeckel+ Co., Hamburg, Germany), with a precision of 0.1 mg.

**Figure 6:**
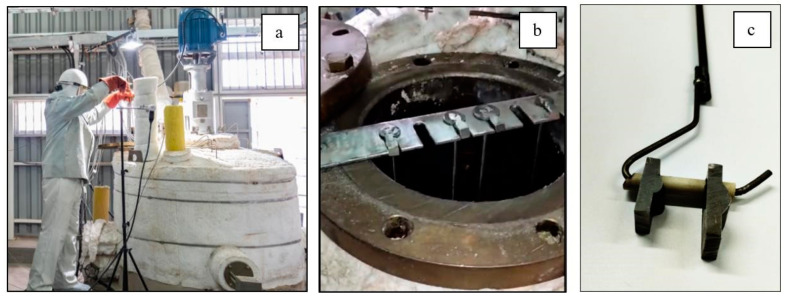
Immersion of samples inside the tank. (**a**) Pilot plant tank with molten salts at 400 °C. (**b**) System for holding stainless steel test tubes and (**c**) test tube rod.

Once the samples were prepared, they were immersed in a thermal storage tank at 400 °C using stainless steel rods 50 cm in height and a metal support plate, where one sample with oscillation welding and one without oscillation were positioned (Figure 6). Throughout this process, the molten salts were kept in circulation to simulate the behaviour of real systems, where the stainless steel is in contact with solar salt in a dynamic system due to the charging and discharging processes of the storage systems.

### 2.6. Metallography and Analysis, Microstructural and Compositional Characterisation

The microstructure was studied through the metallographic analysis following the standard mounting, grinding, and polishing procedures, according to ASTM E3-11. A Mechares 3 mounting machine (PRESI France, Eybens, France) was used for this purpose. It features a fully automated embedding process and complies with ASTM E3 standards. The specimens were ground using sequential-grit-size machines with speed and water flow regulators. The grinding papers adhered to the grinding machine’s plate ranged in grit sizes of 320, 600, and 1200. Before changing papers, any scratches caused by the grinding process were checked using an electronic magnifier, MOTIC model SMZ 171 (PRESI France, Eybens, France).

Similarly, the final polishing of the samples was performed using a Minitech 233 polisher (Sarreguemines, France). This polishing process was sequential, from larger to smaller grain sizes, using wet abrasive grits of 1.0, 0.3, and 0.05 µm on a textile polishing pad. Finally, the specimens were etched in an electrochemical cell using a Vilella reagent (5 mL HCl, 1 g picric acid, and 100 mL ethanol) at a voltage of 4 V. The phases present were then observed under an optical microscope, MOTIC model BA 310 (PRESI France, Eybens, France).

**Figure 7:**
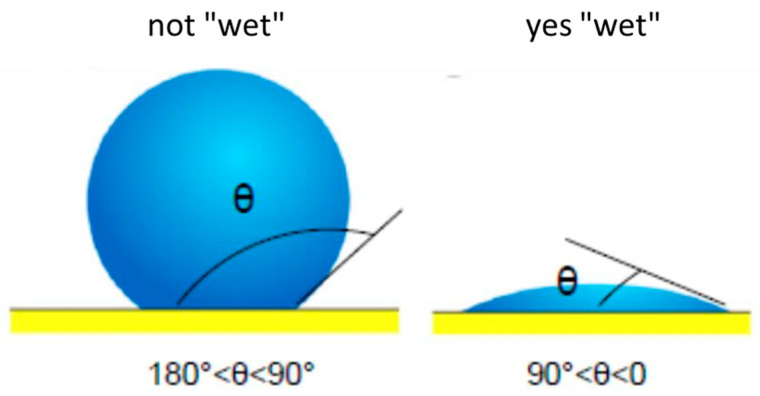
Weld wetting angle.

Dilution in welding is the change in the chemical composition of the metal contribution caused by the mixing that occurs with the metallic base or the previously deposited weld beads. There is a parameter called wetting angle (θ), which refers to the angle formed by the surface of the drop of filler material, upon contact with the substrate. If θ is less than 90°, the drop of filler material tends to wet the surface and the opposite occurs for θ greater than 90° (Figure 7). This indicates that the smaller the contact angle, the better the wetting.

**Figure 8:**
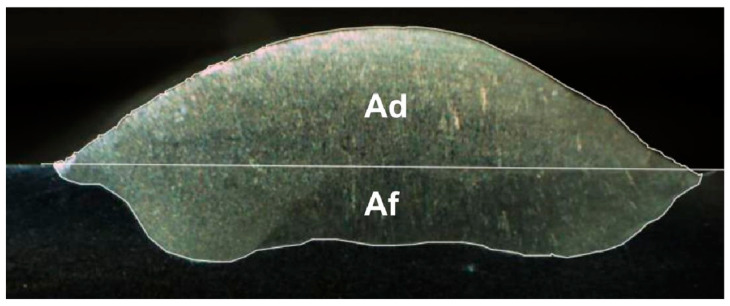
The deposited area ($A_{d}$) and the melted area ($A_{f}$) that make up the cross-section of the bead deposited by the GMAW process.

The dilution coefficient expresses the degree of mixing of the metal used as a filler with the piece to be filled. To determine the dilution of the samples, it is necessary to obtain the macrographic images of the cross-section in the deposited areas ($A_{d}$) and the melted areas ($A_{f}$), according to what is shown in Figure 8.

The following equation determines the degree of dilution: δ is the dilution in percentage, $A_{f}$ is the melted area (mm^2^), and $A_{d}$ is the deposited area (mm^2^).

(4) $$ẟ=\frac{A_{f}}{A_{f}+A_{d}}*100\%$$

The microstructural analysis of the samples was carried out using field emission scanning electron microscopy (FE-SEM) (Itachi High–Technologies, Tokyo, Japan) combined with energy dispersive spectroscopy (EDS). These analyses were conducted at the HERCULES Laboratory in Évora, Portugal, employing a HITACHI S3700N Scanning Electron Microscope (SEM) (Hitachi High–Technologies, Tokyo, Japan) integrated with a BRUKER XFlash 5010 Energy Dispersive Spectroscopy system (Billerica, MA, USA).

## 3. Results and Discussion

### 3.1. Metallography and Material Mass Loss

**Figure 9:**
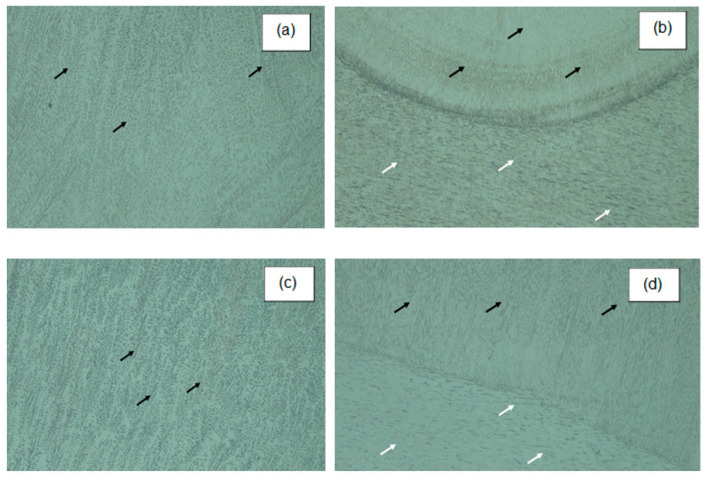
Metallography (50X. Without oscillations: (**a**) In the centre weld, a dendritic phase is shown (black arrows); (**b**) in the HAZ of the weld, two phases are shown, a dendritic phase (black arrows) and an austenitic phase (white arrows). With oscillations: (**c**) In the centre of the weld, a dendritic phase is shown (black arrows); (**d**) in the HAZ zone of the weld, two phases are shown, a dendritic phase (black arrows) and an austenitic phase (white arrows).

Metallographic images obtained using the oscillating arc technique, as shown in Figure 9, reveal smaller and more uniformly distributed dendrites compared to welds performed without this method. The improved distribution and reduced size of these dendrites help mitigate the segregation of impurities such as sulphur and phosphorus at grain boundaries, while also reducing the formation of delta ferrite and chromium carbides in these regions. This occurs because oscillatory welding processes promote a more uniform thermal dissipation, minimising abrupt and localised thermal gradients, which in turn enhances homogeneous dendrite nucleation.

Furthermore, under ISO 5817 [28], the visual inspection (VT) confirmed the absence of discontinuities in the weld seams such as lack of fusion, porosities, and undercuts in both welding processes. This evaluation ensures compliance with the standard’s quality criteria for welded joints.

### 3.2. Microhardness Measurement

**Figure 10:**
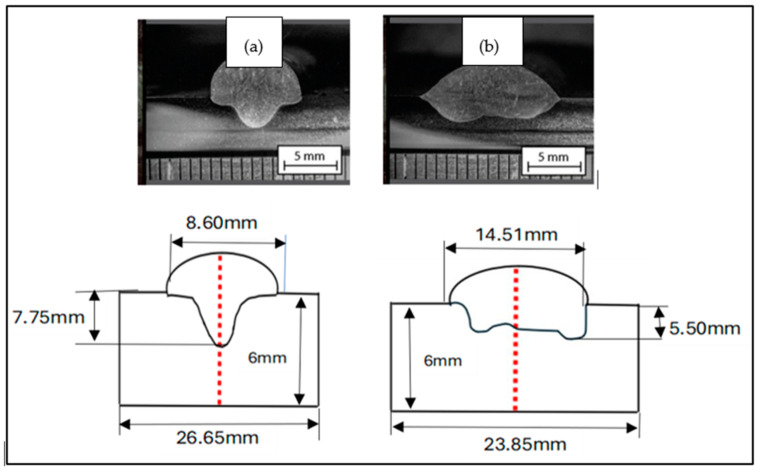
Measurement of transverse microhardness (dotted red line) profile (**a**) without oscillations; (**b**) with oscillations.

The transverse microhardness measurement was performed (below the surface at a distance of approximately 14 microns) under the ASTM E-384 Standard (Vickers microhardness) [29], in a Zwick Roell microhardness tester (Zwick, Ulm, Germany) on the weld bead. The measurement profiles are shown in Figure 10, and the data obtained are presented in Table 6. A lower hardness was obtained in the oscillating arc welds, with an average value of 183.58 HV, compared to 222.21 HV in the non-oscillating specimens. This decrease in microhardness may be mainly associated with the reduction in residual stresses due to arc oscillation.

**Figure 11:**
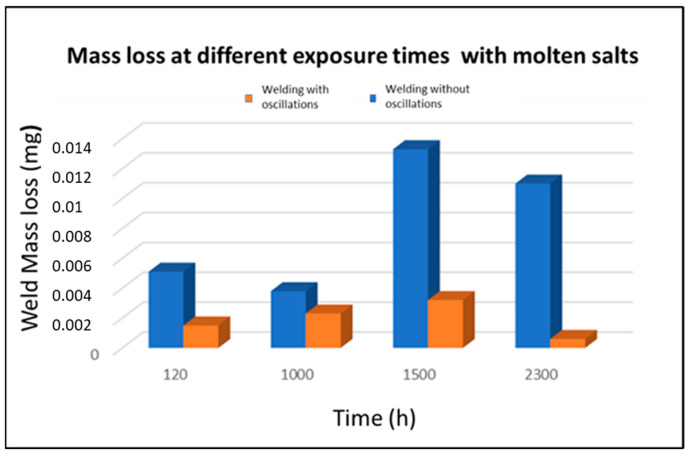
Loss of mass using different welding procedures.

Figure 11 shows the mass loss of the welding samples with and without oscillations from 120 h to 2300 h of exposure in a molten salt medium at 400 °C. The lower mass loss observed in the oscillated samples could be attributed to a more homogeneous distribution of filler material elements in the molten zone, such as chromium and nickel. Furthermore, oscillation welding processes tend to promote more uniform thermal dissipation, as well as localised preheating, due to thermal conduction during each oscillation cycle, which promotes more homogeneous nucleation of dendrites, thus avoiding the overheated spots or excessive melting in certain areas. This promotes more uniform solidification, minimising abrupt thermal gradients that could lead to harmful phases like delta ferrite or chromium carbides at grain boundaries. The presence of these phases can increase the susceptibility of the material to phenomena such as intergranular corrosion. In addition, oscillation welding processes result in longer cooling times due to slower thermal dissipation. Therefore, homogeneous heat distribution and controlled cooling in oscillation welding contribute significantly to the uniformity of the microstructure.

### 3.3. Morphology and Microstructure of Welding

**Figure 12:**
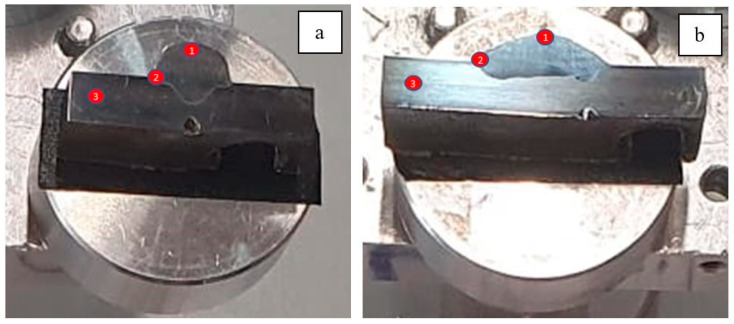
The points analysed with SEM are shown for the sample. (**a**) Without oscillations; (**b**) with oscillations.

This study evaluated the influence of mechanical arc oscillations on the morphology and dilution of weld beads using the GMAW process, comparing welds performed with and without arc oscillations, exposed to a molten salt medium at high temperatures (400 °C). The initial results were analysed using scanning electron microscopy (SEM) analysis after 120, 500, 1000, 1500, and 2300 h of exposure. These analyses focus on the differences in grain morphology, sample mass gain, and the precipitation of phases or compounds. A more detailed analysis of the welding samples with and without oscillation is performed below. Three points of interest were identified within the sample, shown in Figure 12. Point 1 corresponds to the welded filler metal, point 2 corresponds to the heat-affected zone (HAZ), and point 3 corresponds to the base metal of the 316L stainless steel sample.

**Figure 13:**
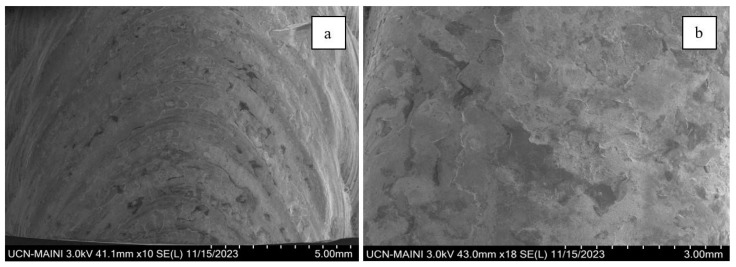
Morphology of corrosion products on the upper surface of 316L stainless steel welded coupons produced using different welding procedures: (**a**) with oscillations and (**b**) without oscillations.

Figure 13 shows the scanning electron microscopy (SEM) analyses of the top view of the two weld beads. A clear difference can be observed between the procedures with and without oscillations. In Figure 13a, a more refined and homogeneous microstructure is evident, suggesting that arc oscillations allow for better heat distribution. This results in finer grains, fewer imperfections, and improved fusion of the filler material with the base material. In contrast, Figure 13b shows that the microstructure exhibits coarser grains and a more irregular morphology without oscillations, likely due to less efficient heat distribution and poorer dilution.

**Figure 14:**
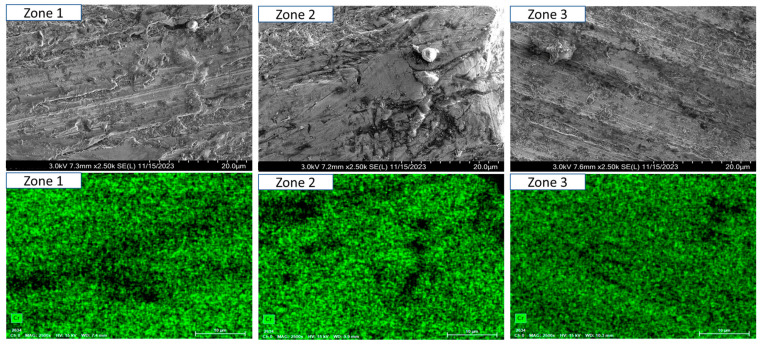
The points analysed with SEM are shown for the weld sample with oscillations at 120 h of exposure with the molten salts: (a) Zone 1, (b) Zone 2, and (c) Zone 3, identified in Figure 12.

**Figure 15:**
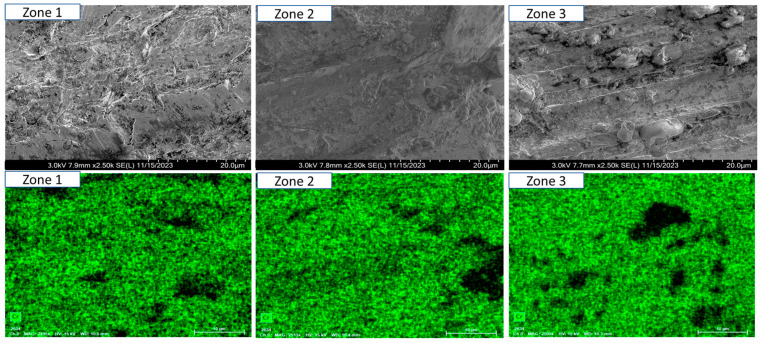
The points analysed with SEM are shown for the weld sample without oscillations at 120 h of exposure with the molten salts: (a) Zone 1, (b) Zone 2, and (c) zone 3, identified in Figure 12.

**Figure 16:**
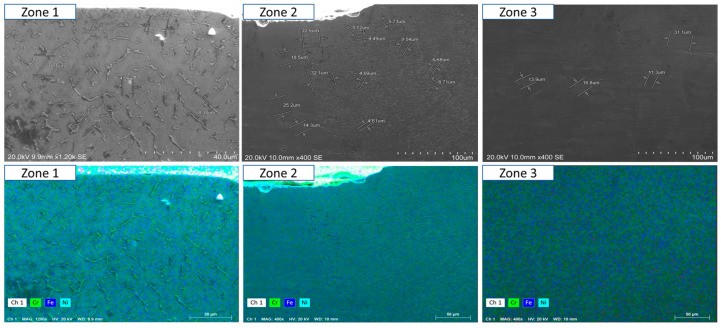
The points analysed with SEM are shown for the weld sample with oscillations at 500 h of exposure with the molten salts: (a) Zone 1, (b) Zone 2, and (c) Zone 3, identified in Figure 12.

**Figure 17:**
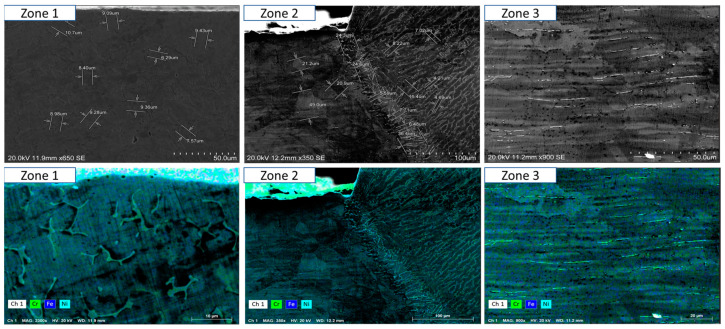
The points analysed with SEM are shown for the weld sample without oscillations at 500 h of exposure with the molten salts: (a) Zone 1, (b) Zone 2, and (c) Zone 3, identified in Figure 12.

**Figure 18:**
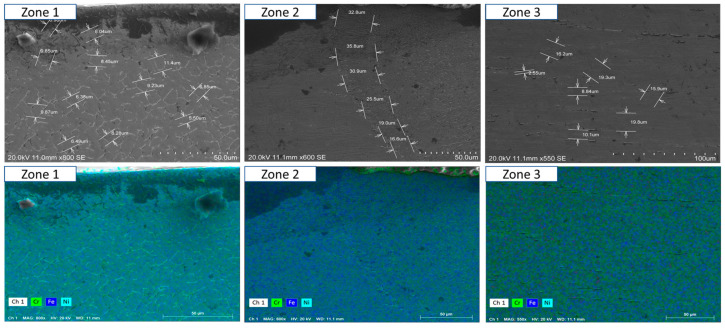
The points analysed with SEM are shown for the weld sample with oscillations at 1000 h of exposure with the molten salts: (a) Zone 1, (b) Zone 2, and (c) Zone 3, identified in Figure 12.

**Figure 19:**
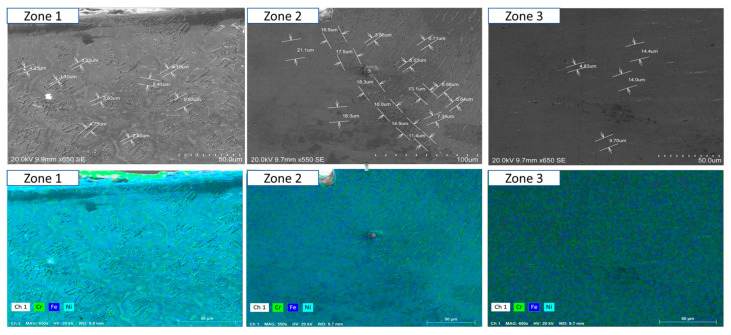
The points analysed with SEM are shown for the weld sample without oscillations at 1000 h of exposure with the molten salts: (a) Zone 1, (b) Zone 2, and (c) Zone 3, identified in Figure 12.

**Figure 20:**
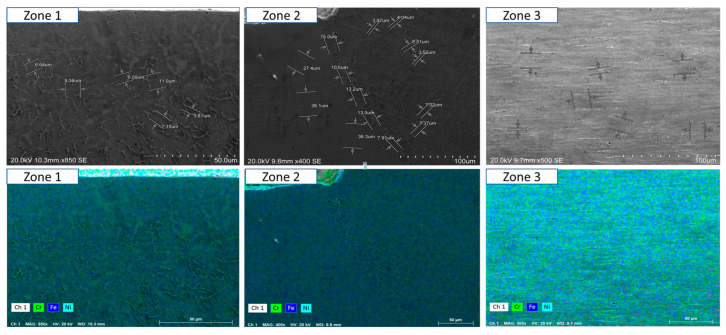
The points analysed with SEM are shown for the weld sample with oscillations at 1500 h of exposure with the molten salts: (a) Zone 1, (b) Zone 2, and (c) Zone 3, identified in Figure 12.

**Figure 21:**
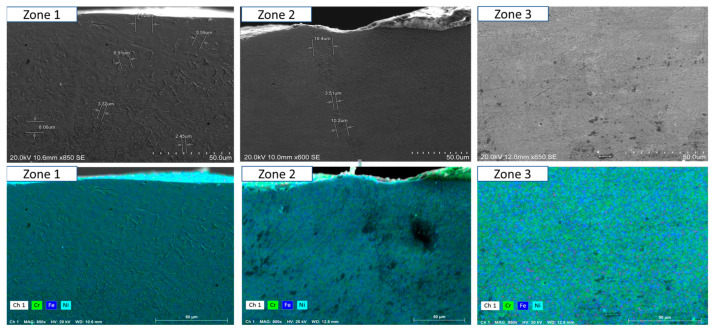
The points analysed with SEM are shown for the weld sample without oscillations at 1500 h of exposure with the molten salts: (a) Zone 1, (b) Zone 2, and (c) Zone 3, identified in Figure 12.

**Figure 22:**
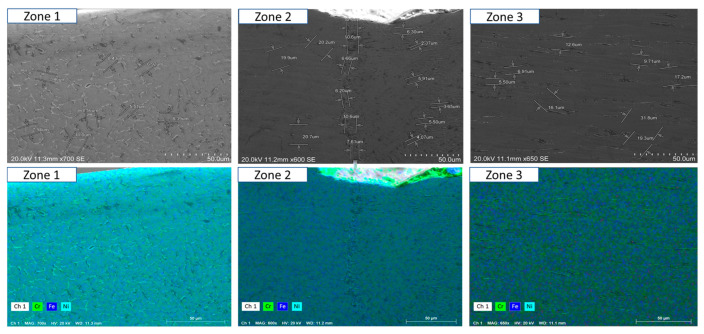
The points analysed with SEM are shown for the weld sample with oscillations at 2300 h of exposure with the molten salts: (a) Zone 1, (b) Zone 2, and (c) Zone 3, identified in Figure 12.

**Figure 23:**
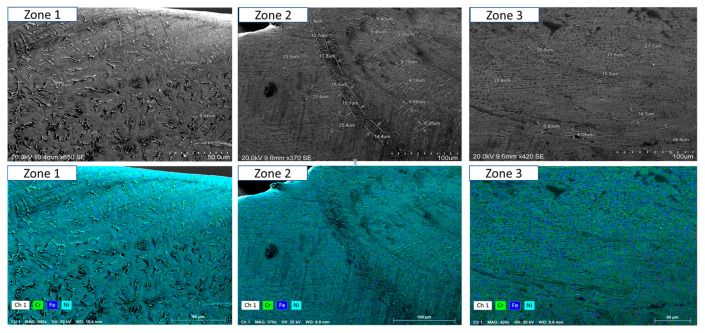
The points analysed with SEM are shown for the weld sample without oscillations at 2300 h of exposure with the molten salts: (a) Zone 1, (b) Zone 2, and (c) Zone 3, identified in Figure 12.

To ensure a clear and detailed examination of the microstructural evolution in 316L steel welds exposed to solar salt at 400 °C for 120, 500, 1000, 1500, and 2300 h, SEM analyses were conducted across different weld zones. Figure 14, Figure 15, Figure 16, Figure 17, Figure 18, Figure 19, Figure 20, Figure 21, Figure 22 and Figure 23 present each condition separately, allowing for a precise assessment of subtle microstructural differences and the influence of oscillation. This approach ensures that critical variations, which might otherwise be obscured in a simplified comparative format, remain clearly distinguishable.

Figure 14 and Figure 15 show the SEM analysis of the oscillated and non-oscillated weld samples after 120 h of exposure to molten salts, with Figure 14 corresponding to the oscillated weld and Figure 15 to the non-oscillated weld. In each figure, the analysis covers: (a) Zone 3 (Base Material), (b) Zone 2 (Heat-Affected Zone), and (c) Zone 1 (Weld Pool). Figure 16 and Figure 17 present the SEM analysis for the 500 h exposure, Figure 18 and Figure 19 for the 1000 h exposure, Figure 20 and Figure 21 for the 1500 h exposure, and Figure 22 and Figure 23 for the 2300 h exposure, following the same format.

Zone 3 (Base Material):

As shown in Figure 14, Figure 15, Figure 16, Figure 17, Figure 18, Figure 19, Figure 20, Figure 21, Figure 22 and Figure 23, Zone 3 represents the base material and remains unchanged during the welding process. Across all the exposure durations (120 h, 500 h, 1000 h, 1500 h, and 2300 h), no significant microstructural changes were observed, regardless of the use of oscillation. This stability confirms the inert nature of the base material under the experimental conditions and isolates the observed effects to the welded regions.

Zone 2 (Heat-Affected Zone, HAZ):

Figure 14, Figure 16, Figure 18, Figure 20 and Figure 22, Zone 2 demonstrate that the HAZ in oscillated welds exhibits a refined and uniform microstructure over time. The use of oscillations promotes improved thermal homogenisation, reducing the thermal gradients and stress concentrations. As a result, the HAZ in oscillated samples shows reduced surface degradation and fewer corrosion products compared to non-oscillated samples. Figure 15, Figure 17, Figure 19, Figure 21 and Figure 23 reveal that the HAZ in non-oscillated samples suffers from significant surface deterioration and higher levels of corrosion. The absence of oscillations leads to pronounced thermal gradients and localised stress, resulting in a microstructure that is more susceptible to molten salt attack.

Zone 1 (Weld Pool):

Figure 14, Figure 16, Figure 18, Figure 20 and Figure 22, Zone 1 show that the weld pool in oscillated samples maintains superior corrosion resistance and a uniform microstructure over the entire exposure duration. Oscillations facilitate the grain refinement mechanisms, including dendrite fragmentation, grain detachment, and heterogeneous nucleation. Those processes result in a denser microstructure with fewer inclusions and voids, which enhances the material’s stability and resistance to degradation. Figure 15, Figure 17, Figure 19, Figure 21 and Figure 23 indicate that the weld pool in non-oscillated samples experiences significant material degradation. Increased porosity, larger corrosion pits, and the accumulation of corrosion products are observed, attributed to the less refined microstructure and residual stresses.

Now, the mechanisms responsible for the enhanced microstructural properties observed in Zones 1 and 2 of oscillation-assisted MIG welds are discussed. Those improvements can be attributed to the three primary grain-refining mechanisms: dendrite fragmentation, grain detachment, and heterogeneous nucleation.

Dendrite fragmentation occurs when the tips of dendrites in the mushy zone are disrupted due to the enhanced convection in the weld pool caused by oscillation. These fragmented dendrites, if stable at the weld pool temperature, act as nuclei for formation of new grains. This mechanism is widely recognised as a common and effective method for grain refinement in weld metals [30].

Grain detachment, closely related to dendrite fragmentation, involves the separation of loosely held grains within the partially melted zone, where the temperature lies between the liquidus (TLiquidus) and eutectic (TEutectic) points. These detached grains, like fragmented dendrites, are carried by the weld pool convection and can serve as nuclei if they remain stable under the prevailing thermal conditions. Although these two mechanisms are sometimes considered distinct, their functional principles are often interlinked.

Heterogeneous nucleation is another key process where atoms in the liquid metal form solid nuclei that exceed the critical size required to overcome the energy barrier for stability. These nuclei can then grow and serve as nucleation sites, promoting a finer and more uniform grain structure [31].

Additionally, the cavitation induced by oscillations enhances nucleation through mechanisms such as locally increasing the melting-point temperature and improving the wetting of the material. As Kou et al. [32] discussed in the context of TIG welding of aluminium alloys, such mechanisms play a pivotal role in forming the refined microstructures, resulting in improved mechanical and corrosion resistance properties.

Applying oscillations in MIG welding amplifies these effects, leading to a more homogeneous and refined microstructure in Zones 1 and 2. This confirms that oscillations during the MIG welding significantly enhance the material properties in these regions, improving overall performance and durability.

General observations regarding the solar salt exposure time can be categorised into two main points:

1. Short-term exposure (120 h and 500 h): The differences between oscillated and non-oscillated welds become apparent in Figure 13 and Figure 15 for oscillated samples and Figure 14 and Figure 16 for non-oscillated samples. Oscillations effectively limit the initial formation of corrosion products and reduce surface irregularities, particularly in Zones 1 and 2.
2. Mid and long-term exposure (1000 h to 2300 h): As shown in Figure 18, Figure 19, Figure 20, Figure 21, Figure 22 and Figure 23, the advantages of oscillations become more pronounced. Oscillated welds demonstrate better structural integrity and resistance to corrosion, while the non-oscillated samples exhibit significant degradation in the HAZ and weld pool.

So, one could conclude that the SEM analysis, presented in Figure 14, Figure 15, Figure 16, Figure 17, Figure 18, Figure 19, Figure 20, Figure 21, Figure 22 and Figure 23, confirms that oscillations during MIG welding significantly enhance the microstructural properties of the HAZ and weld pool under molten salt exposure. The improvements are attributed to grain refinement mechanisms, such as dendrite fragmentation, grain detachment, and heterogeneous nucleation, facilitated by oscillation. These processes result in a more uniform and corrosion-resistant microstructure, reducing the thermal gradients and residual stresses. These findings underscore the critical role of oscillation in extending the durability and reliability of welded structures in harsh environments.

The analysis presented in Section 3.1, Section 3.2 and Section 3.3 confirms that the oscillatory welding technique significantly enhances the mechanical integrity, microstructural homogeneity, and corrosion resistance of welded joints. The findings demonstrate that oscillated welds exhibited a wider bead with a more uniform and refined microstructure (Figure 1). Compared to non-oscillated welds, the oscillated arc promoted grain refinement due to a more controlled heat input and reduced cooling rates.

This refinement enhances mechanical properties and reduces hardness variations across the weld. Additionally, oscillated welds demonstrated a more uniform grain structure, as the oscillating motion contributed to a more balanced heat distribution. Unlike non-oscillated welds, the controlled thermal cycles not only refined grain size but prevented excessive growth as well, ensuring a more stable mechanical structure. This microstructural refinement is further reflected in the metallographs obtained using the oscillating arc technique (Figure 9), which reveal smaller and more uniformly distributed dendrites compared to welds performed without this method.

The improved distribution and reduced size of these dendrites help mitigate the segregation of impurities such as sulphur and phosphorus at grain boundaries, while also reducing the formation of delta ferrite and chromium carbides in these regions. This occurs because oscillatory welding processes promote a more uniform thermal dissipation, minimising abrupt and localised thermal gradients, which, in turn, enhances homogeneous dendrite nucleation. These microstructural modifications directly contribute to increased mechanical stability and corrosion resistance, making oscillated welds more reliable in demanding environments.

The parent material analysis demonstrates that, since the oscillated arc disperses the heat input over a broader area, it minimises the thermal influence on the parent material. This effect helps maintain the original microstructure and mechanical properties of the base metal, reducing residual stress accumulation and minimising the risk of distortion and cracking. The controlled heat application in oscillated welding ensures that the parent material experiences a lower peak temperature, reducing the likelihood of microstructural degradation and thermal expansion issues.

The analysis of the HAZ shows that oscillations altered the thermal cycle, generating a more gradual temperature gradient. This effect reduces the risk of excessive grain growth, which is common in conventional welding due to localised overheating. As a result, the HAZ in oscillated welds exhibited lower hardness values and a smoother transition between the weld metal and the parent material, which potentially enhances toughness and reduces susceptibility to cracking. Additionally, the improved heat distribution contributed to a reduction in thermal stresses within the HAZ, leading to improved microstructural stability.

### 3.4. Geometric Analysis of Welds

**Figure 24:**
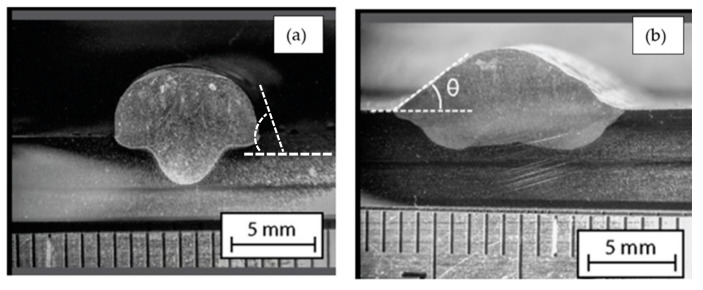
The wet angle of the weld (**a**) without oscillations and (**b**) with oscillations.

**Figure 25:**
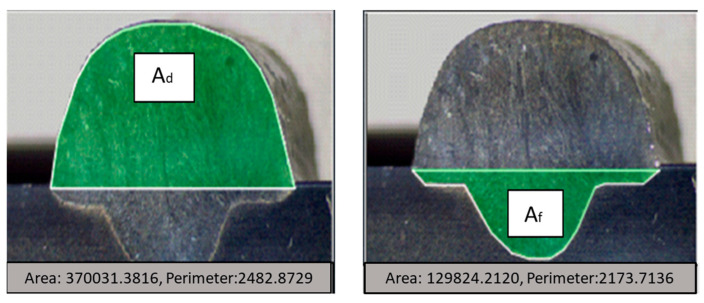
The degree of dilution for the samples without oscillations.

**Figure 26:**
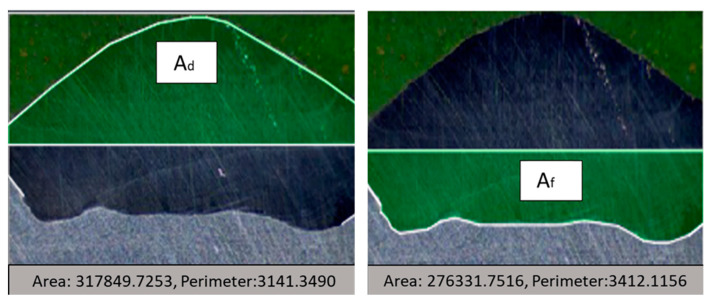
The degree of dilution for the samples with oscillations.

The wetting angle and the degree of dilution determine the quality of the weld bead. In Figure 24, it is possible to observe the difference in the wetting angle for the sample with and without oscillation. Figure 24a shows that the angle of the sample without oscillations is between 77 and 73°, and in Figure 24b the angle of the sample with oscillations fluctuates between 35 and 29°. The degrees of dilution are presented in Figure 25 and Figure 26. Figure 25 illustrates the dilution for samples without oscillation, where an $A_{f}$ area of 129,824 mm^2^ and an $A_{d}$ area of 370,031 mm^2^ were obtained, resulting in a dilution degree of 74%. In contrast, for the sample with oscillation (Figure 26), an $A_{f}$ area of 276,332 mm^2^ and an $A_{d}$ area of 317,850 mm^2^ were measured, leading to a dilution degree of 53.5%.

In the case of the welded samples with oscillations, a significantly lower wetting angle of 30 to 35° was recorded, compared to the 75° observed in the welds without oscillations. The penetration was lower, reaching only 5.5 mm compared to the 7.75 mm achieved in the welds without oscillations (Figure 10). These last specimens presented a narrow and deep penetration profile, known as a “finger shape”, as shown in Figure 24a. The characteristics observed in swing welds are particularly beneficial in the last passes of the weld beads, as they allow larger areas to be covered efficiently.

The results indicate that the lower dilution factor observed in swing welds ensures that the filler material predominates in the weld zone. This aspect is crucial in stainless steel, where the designed chemical composition of the filler material is a key to guarantee the corrosion resistance. In this case, a solid ER 316L wire was used, the formulation of which minimises the interaction with the alloying elements of the base metal. This behaviour is essential to maintain the chemical integrity of the deposited metal, increasing its durability and resistance to corrosive environments.

## 4. Conclusions

The results obtained in this study confirm that oscillating arc welding significantly improves the microstructural properties and corrosion resistance of welded joints exposed to high-temperature molten salts.

Metallographic analysis revealed that the application of arc oscillations produced smaller dendrites with a more uniform distribution than the non-oscillated process. This microstructural modification reduced the segregation of impurities, such as sulphur and phosphorus, at grain boundaries, minimising the formation of undesired phases such as delta ferrite and chromium carbides.

Immersion tests in molten salts at 400 °C showed a lower mass loss in the oscillated welded samples, which is associated with a lower degree of dilution. This phenomenon favoured a more homogeneous distribution of alloying elements, such as chromium and nickel, in the fusion zone, promoting more uniform solidification and reducing the formation of abrupt thermal gradients.

The geometric analysis of the weld beads showed that the oscillations process resulted in a lower wetting angle and a lower degree of dilution than welding without oscillation. These characteristics enhance the coverage and distribution of the filler material, ensuring that its designed chemical composition predominates in the fusion zone, which is crucial for improving corrosion resistance in molten salt environments.

Additionally, microhardness measurements indicated that the application of arc oscillations significantly reduced residual stresses in the welded area, decreasing the susceptibility to stress corrosion cracking. The phase evolution in the weld zone and the heat-affected zone (HAZ) did not show significant modifications after exposure to molten salts throughout the entire test period.

In conclusion, oscillating arc welding emerges as a promising alternative to improve the quality and durability of welded joints in thermal energy storage systems using molten salts. However, further studies at higher temperatures and with extended exposure times are recommended to assess its long-term performance under more demanding operating conditions, particularly in thermal storage tanks for concentrated solar power (CSP) plants.

## Figures and Tables

**Table 1:** AISI 316L steel composition, wt%.

AISI	C	Mn	Si	Cr	Ni	Mo	P	S
316L	0.039	1.14	0.393	16.54	10.30	1.99	0.024	0.0024
Standard	0.03	2.00	0.75	16–18	10–14	2–3	0.045	0.030

**Table 2:** Constant parameters for both weld seams.

Oscillation amplitude A (mm)	12
Reference voltage Ur (V)	18
Oscillation type	Triangular
Welding speed (cm/min)	15
Shielding gas type	80% Ar + 20% CO_2_
Flow rate (L/min)	16
Welding position	Flat
Transfer type	Short Circuit
Nozzle-to-work distance DTP (mm)	15
Wire type	AWS ER316L
Wire diameter (mm)	1.2

**Table 3:** Variable parameters for weld beads.

Welding Bead Type	Oscillations Type	Frequency (Hz)	Wire Speed (m/min)
Without oscillations	N/A	0	4.0
With oscillations	Triangular	1	4.0

**Table 4:** Speed, welding current, and energy.

Test	$V_{resul}$ (mm/min)	$I_{w}$ (A)	$E_{w}$ (KJ/mm)
Without oscillations	150	145	0.960
With Oscillations	735	143	0.188

**Table 5:** Filler material characteristics (FW).

Description	Composition	(% wt)
Standard Specification	C	0.02
AWS	Mn	1.69
AWS ER-316 L	Si P	0.38 0.03
Material	S	0.008
Stainless steel (ASS)	Cr Ni Mo	18.1 11.1 2.1

**Table 6:** Microhardness measurement.

Item	Hardness HV (Without Oscillations)	Hardness HV (With Oscillations)
1	233	188
2	222	181
3	237	192
4	241	188
5	229	192
6	237	183
7	225	176
8	230	173
9	221	172
10	206	180
11	202	188
12	200	190
13	203	
14	205	
15	210	
Average	220	184

## Data Availability

The original contributions presented in the study are included in the article; further inquiries can be directed to the corresponding author.
